# Investigation of Ki-67 and Clinical Outcome in Feline Nasal Adenocarcinoma Treated Using Hypofractionated Radiotherapy

**DOI:** 10.3390/ani14243573

**Published:** 2024-12-11

**Authors:** Premkamon Aonta, Piraya Jaiyangyeun, Wutthiwong Theerapan, Supreeya Srisampan, Charuwan Wongsali, Attawit Kovitvadhi, Tassanee Jaroensong

**Affiliations:** 1 Department of Companion Animal Clinical Sciences, Faculty of Veterinary Medicine, Kasetsart University, 50 Ngamwongwan Rd., Lat Yao, Chatuchak, Bangkok 10900, Thailand; premkamon.ao@ku.th (P.A.); wutthiwong.t@ku.th (W.T.); 2 Kasetsart Veterinary Imaging and Radiotherapy Center, Faculty of Veterinary Medicine, Kasetsart University, 50 Ngamwongwan Rd., Lat Yao, Chatuchak, Bangkok 10900, Thailand; fvetpaj@ku.ac.th; 3 Center for Veterinary Diagnostic Laboratory-Bangkhen, Faculty of Veterinary Medicine, Kasetsart University, 50 Ngamwongwan Rd., Lat Yao, Chatuchak, Bangkok 10900, Thailand; yyungvet@gmail.com (S.S.); fvetcrw@ku.ac.th (C.W.); 4 Department of Physiology, Faculty of Veterinary Medicine, Kasetsart University, 50 Ngamwongwan Rd., Lat Yao, Chatuchak, Bangkok 10900, Thailand; fvetawk@ku.ac.th

**Keywords:** cats, hypofractionated radiotherapy, Ki-67, nasal adenocarcinoma, prognosis

## Abstract

Ki-67 has been reported as a prognostic marker in human cancers treated using RT. The current study investigated the prognostic significance of Ki-67 expression and its association with clinicopathological characteristics in 19 cats diagnosed with nasal adenocarcinoma and treated using hypofractionated RT. Data collected encompassed signalment, clinical signs, clinicopathological variables, treatment outcomes, and survival times. Median survival times (MST) were estimated using Kaplan–Meier curves and analyzed based on the log-rank test. Based on the results, white blood cell counts were significantly (*p* < 0.001) higher before treatment than after, although this was not associated with survival time. The overall MST was 550 days (range: 56–1118 days). Cats achieving a favorable response (complete or partial response) had significantly (*p* = 0.006) longer survival times (1055 days) than those with a poor response (stable or progressive disease; 369 days). Cats with high Ki-67 expression had significantly (*p* = 0.028) longer survival times (1055 days) than those with low Ki-67 expression (256 days). These results suggested that Ki-67 may be a potential prognostic factor for feline nasal adenocarcinoma.

## Simple Summary

Nasal adenocarcinoma is the second most common nasal tumor in cats, and radiotherapy (RT) is a common treatment. Ki-67, a prognostic marker in human cancers treated using RT, was investigated in this retrospective study of 19 cats with nasal adenocarcinoma that had been treated using hypofractionated RT. Based on the results, white blood cell counts were significantly higher before treatment than after. Cats with a favorable response to treatment had significantly longer survival times than those with a poor response. Additionally, cats with high Ki-67 expression had significantly longer survival times than those with lower Ki-67 levels.

## 1. Introduction

Nasal tumors comprise 1–8.4% of all feline tumors, with over 90% being malignant [1,2]. Adenocarcinomas represent the second most common type of tumor within the feline nasal cavity [3,4,5], typically affecting older, feline leukemia virus (FeLV)-negative and feline immunodeficiency virus (FIV)-negative individuals [6,7]. Clinical manifestations commonly include nasal or ocular discharge, upper respiratory tract noise, sneezing, dyspnea, epistaxis, facial deformity, and weight loss [5,7,8,9]. Nasal carcinomas are characterized by local invasiveness and a low metastatic rate at the time of diagnosis [5,10].

Radiotherapy (RT) is widely considered the treatment of choice for nasal carcinoma in cats since surgical options are limited due to the inability to achieve complete excision, and chemotherapy alone is generally ineffective for most nasal tumors [9,10]. RT treatment for feline nasal carcinoma has been investigated in a few studies [6,7,9]. Survival times have been reported of 360–721 days using definitive RT [6,9,11] and 284–450 days using hypofractionated RT [3,7,11,12].

Ki-67 is a non-histone nuclear protein expressed in actively proliferating cells during all cell cycle phases except G0, the resting phase [13]. Several studies in human oncology have demonstrated that high levels of Ki-67 are associated with better responses to RT than with tumors with lower Ki-67 [14,15,16]. In veterinary oncology, Ki-67 has been investigated in various types of feline cancer [17,18,19]. Based on the results of one study of feline nasal lymphoma, cats with high Ki-67 expression (>40%) had longer survival times and responded more favorably to RT than cats with lower Ki-67 expression [19]. Another study reported that nasal and periocular squamous cell carcinomas with low Ki-67 levels had a significantly shorter disease-free interval than tumors with high Ki-67 levels [18]. However, several studies in both human and veterinary medicine have considered high Ki-67 expression as a generally unfavorable prognostic factor [20,21,22,23].

Few studies have focused on prognostic factors for RT in veterinary medicine, and specifically, to the authors’ knowledge, no studies have yet investigated the role of Ki-67 in feline nasal adenocarcinoma. Therefore, the objective of this study was to investigate the prognostic significance of Ki-67 expression and clinicopathological characteristics in cats with nasal adenocarcinoma and treated using hypofractionated RT.

## 2. Materials and Methods

### 2.1. Case Selection

Inclusion criteria for the study were cats with histologically diagnosed nasal adenocarcinoma that had been treated using hypofractionated RT at the Kasetsart University Veterinary Teaching Hospital, Bangkok, Thailand, between July 2018 and December 2023. All cats had samples collected via rhinoscopic biopsy. None of the cats exhibited metastasis to the mandibular lymph node, as determined by fine needle aspiration for cytology or computed tomography (CT), nor to the lungs, as assessed by thoracic X-ray or CT. Tumors of the nasal planum were excluded from the study. Each patient was evaluated for age, gender, breed, body weight, reproductive status, clinical signs, retroviral status, blood profile, tumor staging, tumor response, adverse events, and additional chemotherapy. Tumor staging was based on CT scans using the modified Adams staging system [24], categorized as follows: stage T1, confined to one nasal passage, paranasal sinus, or frontal sinus, with no bone involvement beyond the turbinates; stage T2, any bony involvement beyond the turbinates, but without evidence of orbital, subcutaneous, or submucosal masses; stage T3, orbital involvement or the presence of nasopharyngeal, subcutaneous, or submucosal masses; and stage T4, tumor causing lysis of the cribriform plate. Follow-up data were obtained from medical records or by contacting the veterinarian or owner.

### 2.2. Radiotherapy and Response Assessment

Prior to RT, all cats underwent a CT scan (Optima CT660, GE Healthcare, Milwaukee, WI, USA) for tumor staging based on the Adams staging system and for CT-based treatment planning. RT was delivered using a 6 MV linear accelerator (Varian Clinac 2100C, Varian Medical Systems Inc., Palo Alto, CA, USA) following a hypofractionated protocol of 6 Gy per fraction, delivered once weekly, for a total of 6 to 8 fractions.

The planning target volume (PTV) was defined as the region extending 0.5 cm beyond the gross tumor volume (GTV), while the clinical target volume (CTV) was expanded by 0.2 cm. Radiation plans were developed using intensity-modulated radiation therapy with the goal of delivering 100% of the prescribed dose to 100% of the CTV and 95% of the prescribed dose to the PTV. Beam angles were carefully adjusted to avoid irradiating the eyeballs, ensuring that optimal coverage was confined to the tumor area. Throughout the treatment process, all cats were anesthetized and positioned in sternal recumbency. The head was elevated using cushions, while a mouth gag was placed in the maxilla to achieve a more even surface between the nose and the frontal sinus, thereby reducing the radiation dose to the tongue.

Tumor response was assessed 1 month after treatment by measuring the tumor volume before and after RT. Tumor volume was manually outlined in the CT image on a slide-by-slide basis and semi-automatically calculated using software (AW 4.6 workstation, GE Healthcare, Milwaukee, WI, USA). The percentage change in tumor volume was calculated by comparing the initial tumor volume to the volume at 1 month after treatment. Based on volumetric criteria, the cats were classified into four response groups: complete response (CR) with complete tumor disappearance, partial response (PR) with more than 60% tumor volume reduction, stable disease (SD) with more than 40% tumor volume reduction, and progressive disease (PD) for those not meeting any of the other three criteria [25,26,27].

### 2.3. Clinicopathological Evaluations

The blood profile was evaluated using RT at the initial visit (before treatment) and again 1–2 months after treatment. The hematological parameters evaluated were hematocrit (HCT) and white blood cell count (WBC), which were measured using a Sysmex XN-1000™ Hematology Analyzer (Sysmex, Mundelein, IL, USA). The serum biochemistry parameters assessed were serum creatinine (sCr) and alanine aminotransferase (ALT) and were determined using an IL Lab 650 chemistry system (Diamond Diagnostics, Holliston, MA, USA). Screening for retroviral infections (FeLV antigen and FIV antibody) was performed using a rapid immune migration-based point-of-care test kit (WITNESS^®^ FeLV-FIV, Zoetis, Lyon, France).

Adverse events were classified as early effects if they occurred within <3 months RT and as late effects if they occurred ≥3 months after RT. Grading of radiation adverse effects was conducted retrospectively from the medical records using the Veterinary Radiation Therapy Oncology Group (VRTOG) toxicity criteria [28].

### 2.4. Immunohistochemistry Staining

**Figure 1:**
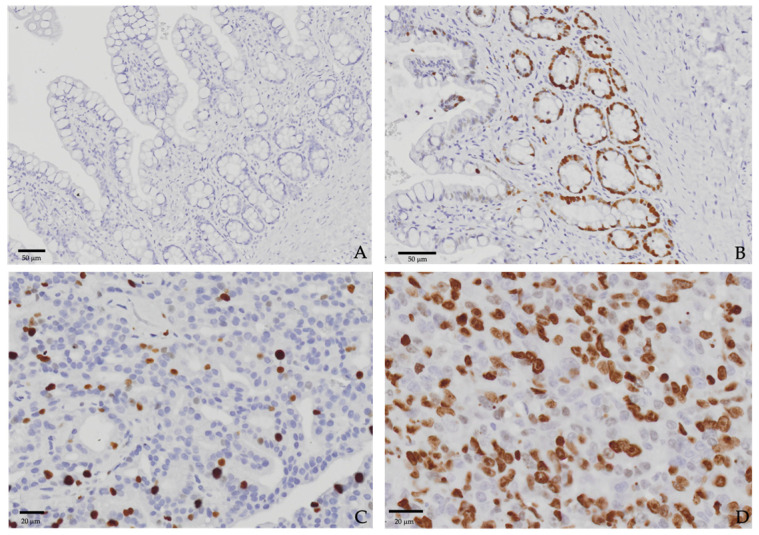
Immunohistochemical staining of feline intestinal mucosa. (**A**) Negative control and (**B**) positive control. Immunohistochemical staining of nasal adenocarcinoma tissue. (**C**) Tumor tissue with low Ki-67 expression (9.59%), stage T3, and (**D**) tumor tissue with high Ki-67 expression (65.07%), stage 4. Original magnification, 600×.

All tumor tissues were obtained based on incisional biopsy, fixed in 10% neutral buffer formalin, and embedded in paraffin. Sections (3 μm) were stained using hematoxylin and eosin (H&E). The H&E-stained sections were evaluated by Thai Board-accredited pathologists to diagnose and characterize the morphological descriptions. For immunohistochemistry staining after deparaffinization and rehydration, antigen retrieval involved immersion in a citrate-based buffer containing surfactant (pH 6.0) for 45 min in a vegetable steamer. All sections were blocked for endogenous peroxidase activity and non-specific binding using 3% hydrogen peroxide and 0.4% casein in phosphate-buffered saline (PBS), respectively. Then, the sections were incubated using a mouse anti-Ki-67 monoclonal antibody (clone MIB-1, Dako, Glostrup, Denmark) at a 1:100 dilution in PBS for 60 min at room temperature. The secondary antibody dilution and avidin–biotin complex solution were applied to the sections and incubated for 30 min each at room temperature. Immunolabelling was performed using Novolink Polymer detection (Leica Biosystems, NCL, Newcastle upon Tyne, UK). Finally, the reactions were visualized using DAB as a chromogen; sections were counterstained with hematoxylin. A normal feline intestinal mucosa was used as the positive control, whereas the negative control, as shown in Figure 1, was conducted on slides without the primary antibody [29]. The Ki-67 index was determined by calculating the percentage of Ki-67-positive tumor cells out of 1000 cells, counted across 5 random high-power fields (600×) under microscopic examination. Areas with severe inflammation or necrosis were excluded from the analysis. The Ki-67 index was divided into two groups (high and low Ki-67), with 40% used as the cutoff point, as reported in another study [19].

### 2.5. Statistical Analysis

The overall survival time was defined as the number of days from the initiation of RT until death from any cause. Cases were censored if the individual was alive at the time of analysis or lost to follow-up. The correlation between median survival time (MST) and prognostic factors was estimated using Kaplan–Meier curves, with statistical differences between survival curves being calculated using the Cox proportional hazards model. Differences in Ki-67 levels between favorable response groups (CR and PR) and poor response groups (SD and PD) were analyzed using the Mann–Whitney test. Paired pre- and post-treatment blood profile results were compared using the Wilcoxon signed-rank test. A value of *p* < 0.05 was used to test for statistical significance.

All statistical analyses were performed using RStudio (Version 2023.12.1, https://rstudio.com/products/rstudio/download/ (accessed on 17 July 2024))—an integrated development environment for R, which is a programming language for statistical computing and graphics (R Core Team, 2007). Survival analysis was conducted using the survival package for Cox proportional hazards regression and survival curve fitting, whereas the ‘survminer’ package was used for visualization of survival curves. Hazard ratios were calculated using a value of *p* < 0.05 to test for statistical significance.

## 3. Results

In total, 49 cats were histologically diagnosed with nasal adenocarcinoma, of which 19 met the inclusion criteria. The characteristics of these 19 cats are presented in Table 1. Of these 19 cats, 8 were male (neutered: *n* = 6, intact: *n* = 1, unknown: *n* = 1) and 11 were female (neutered: *n* = 7, intact: *n* = 2, unknown: *n* = 2). The cats were aged 2–17 years, with a median age of 10 years. Body weights were in the range of 2.5–6.6 kg, with a median weight of 4.27 kg. The study predominantly included domestic shorthair (DSH) cats (*n* = 16, 84.21%), with two Scottish Folds and one Persian. The FeLV and FIV serology results were 2 cats (10.53%) tested positive for FeLV and 13 cats (68.42%) tested negative for both infections. Data for four cats were unavailable. At the time of diagnosis, the most prevalent clinical signs were epistaxis (*n* = 13, 68.42%) and facial deformity (*n* = 13, 68.42%). These were followed by sneezing (*n* = 12, 63.16%), nasal discharge (*n* = 7, 36.84%), dyspnea (*n* = 5, 26.32%), submandibular lymph node enlargement (*n* = 3, 15.79%), ocular discharge (*n* = 3, 15.79%), and upper respiratory noise (*n* = 3, 15.79%). According to the modified Adam’s staging system for canine nasal tumors, the cats were classified into stages T2 (*n* = 1, 5.26%), T3 (*n* = 8, 42.11%), and T4 (*n* = 10, 52.63%).

The mean total radiation dose administered was 45.79 Gy, with a median dose of 48 Gy and a range of 36 to 48 Gy. Following RT, two cats were in the response group CR, eight were in PR, three were in SD, and four were in PD. No post-RT CT scan data were available to evaluate tumor response in two cats: one cat experienced cardiac arrest immediately after completing the last session of RT, while the second cat was lost in the follow-up process. Five cats received a second course of hypofractionated RT, with a median total dose of 48 Gy (range: 36–48 Gy) and a mean total dose of 43.2 Gy. The cause of death was documented in the medical records: one cat died from aspiration pneumonia and pancreatitis (this cat had an oronasal fistula from the mass), two from seizures, two from renal failure, and five from unknown causes. At the time of data analysis, seven cats were still alive.

Acute adverse events were observed in 12 of the 19 cats (63.16%). Specifically, seven cats developed grade 1 alopecia, five cats experienced conjunctivitis (one with VRTOG grade 1 and four with grade 2), and one cat developed oral mucositis of unknown grade. Late adverse events were not recorded in the medical records.

Ki-67 immunostaining, as shown in Figure 1, was performed on all 19 cats. The percentage of Ki-67-positive tumor cells was in the range of 6.17–70.10%, with a median of 41.42% and a mean of 43.33%. Seven cats (36.84%) were categorized as having low Ki-67 levels, while twelve cats (63.16%) were classified in the high Ki-67 group.

The median Ki-67 indices for the different response groups were as follows: CR group: 58.82% (range: 54.49–63.15%), PR group: 48.23% (range: 29.76–65.07%), SD group: 22.52% (range: 6.17–55.36%), and PD group: 35.89% (range: 30.01–70.01%). When combining the CR and PR groups, the median Ki-67 index was 54.76% (range: 29.76–65.07%), whereas the SD and PD groups had a median of 35.34% (range: 6.17–70.10%). The difference in Ki-67 indices between the favorable response groups (CR and PR) and the poor response groups (SD and PD) was not statistically significant (*p* = 0.133).

The hematological and serum biochemistry data for this study were obtained from medical records, as shown in Table 2. Before treatment, the median HCT of the cats was 32.6% (range: 27.3–46.3%), the median WBC was 19.36 × 10^3^ cells/μL (range: 8.83–117.73 × 10^3^ cells/μL), the median sCr was 1.61 mg/dL (range: 1.21–2.58 mg/dL), and the median ALT was 41 IU/L (range: 25–163 IU/L). After treatment, the median HCT of the cats was 35% (range: 25.30–38.30%), the median WBC was 14.39 × 10^3^ cells/μL (range: 5.8–37.92 × 10^3^ cells/μL), the median sCr was 1.67 mg/dL (range: 1.16–2.89 mg/dL), and the median ALT was 46 IU/L (range: 22–83 IU/L). There were no significant differences in the HCT, sCr, and ALT levels, whereas the WBC was significantly (*p* < 0.001) lower after treatment compared to before treatment.

**Figure 2:**
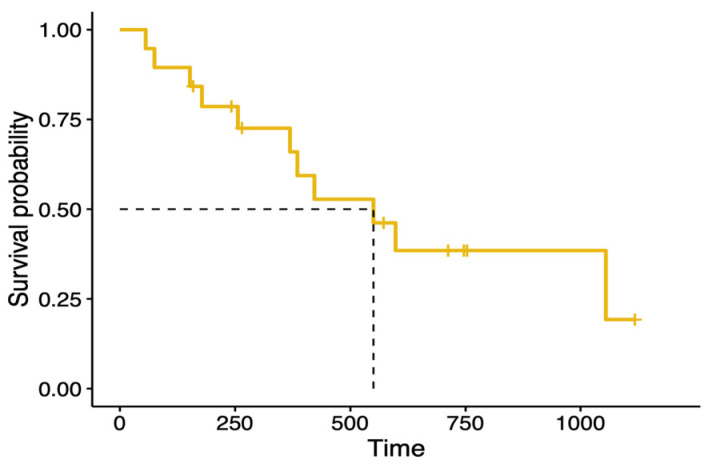
Kaplan–Meier survival curve showing overall median survival of 19 cats with nasal adenocarcinoma treated using hypofractionated RT. The MST was 550 days (range 56–1118 days).

Based on the survival analysis of the 19 cats, the overall MST was 550 days, with a range of 56–1118 days, as shown in Figure 2. Cats that achieved a CR or PR had significantly (*p* = 0.006) longer survival times (1055 days) compared to those with SD or PD (369 days).

**Figure 3:**
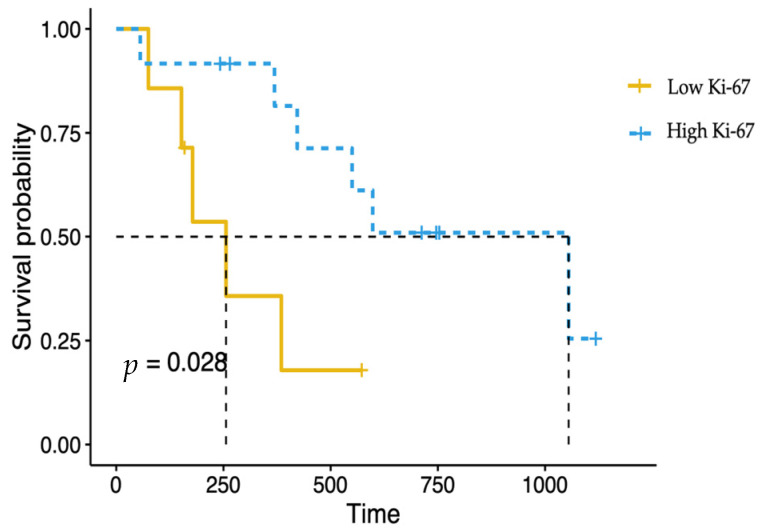
Log-rank test analysis of survival times based on Ki-67 expression levels. Cats with high Ki-67 levels (MST: 1055 days; yellow line) had significantly (*p* = 0.028) longer survival times than cats with low Ki-67 levels (MST: 256 days; blue dashed line).

The MST was evaluated across various factors: gender, age, tumor stage, body weight, clinical signs (sneezing, epistaxis, facial deformity, nasal discharge, dyspnea, ocular discharge, upper respiratory noise, submandibular lymph node enlargement), a hematological parameter, a serum biochemistry parameter, radiation dose, and additional RT. However, according to the log-rank test analysis, none of these factors had a significant difference in MST. Notably, cats with high Ki-67 expression had significantly (*p* = 0.028) longer survival times (1055 vs. 256 days) compared to those with low Ki-67 expression, as illustrated in Figure 3.

## 4. Discussion

This study demonstrated that nasal adenocarcinoma predominantly occurred in older cats, with a median age of 10 years, consistent with findings from other studies [4,9,11]. Most of the cats in the current study tested negative for FeLV and FIV, which was also consistent with other reports [6,7]. Notably, DSH cats were overrepresented, as observed in another study [12], accounting for more than 80% of the cases in the current study. This high prevalence of DSH cats may have been influenced by the geographical location of the study; however, no specific data were collected on breed distribution, making it difficult to draw any definitive conclusions about breed predisposition. The most prevalent clinical signs at the time of diagnosis were epistaxis, facial deformity, sneezing, and nasal discharge, which are common in nasal tumors [3,5,7,8,9,11]. Most cats in this study had advanced-stage disease, classified as stage 3 or 4 according to the modified Adams system, which was consistent with another report [9]. The reasons for presentation at such advanced stages are unclear. It could have been that cats with nasal tumors are initially misdiagnosed with chronic rhinitis, leading to delays in further diagnostic workup, including advanced imaging. Additionally, some owners may not have recognized the clinical signs in their cats early on.

Based on the results from the current study, the overall MST was 550 days, which was longer than reported elsewhere [9,12]. Other studies identified several prognostic indicators for feline nasal tumors, including anemia, radiation dose, treatment response, cribriform plate destruction, epistaxis, facial deformity, and the Adams-modified stage IV classification [9,12,30,31]. However, in the current study, none of the evaluated prognostic factors were predictive of outcome, with the exception of tumor response and Ki-67.

Ki-67 is a marker of cell proliferation, expressed in actively dividing cells during all phases of the cell cycle except G0, and is used to indicate how rapidly cells are dividing. High Ki-67 levels are typically associated with more aggressive tumors, as these rapidly dividing cells have a higher tendency to invade nearby tissues, metastasize, and resist certain therapies [32,33]. Therefore, elevated Ki-67 levels are often linked to a poor prognosis, as reported in previous studies on feline oral squamous cell carcinomas [20], canine mast cell tumors [22], and cancers of the glottis, oropharynx, and oral cavity in humans [21,23]. However, in some cases, particularly with RT, high Ki-67 levels may correlate with a better prognosis, as seen in feline nasal and periocular squamous cell carcinomas [18] and feline nasal lymphoma [19]. This is because RT targets rapidly dividing cells, making tumors with high proliferation rates more vulnerable to the treatment. RT damages the DNA of fast-replicating cells, causing tumor shrinkage and improving outcomes [34]. Thus, while high Ki-67 usually signifies aggressiveness, it may also indicate increased radiosensitivity, leading to a favorable response to RT.

In the current study, cats that showed a favorable response (CR and PR groups) had higher Ki-67 levels (54.76%) compared to those with a poor response (SD and PD groups) (35.34%), although this difference was not statistically significant (*p* = 0.133). Moreover, cats with high Ki-67 levels had a significantly longer median survival time (MST) than those with lower levels (1055 vs. 256 days, respectively; *p* = 0.028). This finding is consistent with another report on feline nasal lymphoma [19], which demonstrated that both Ki-67 and the apoptotic index were strong predictors of response to RT in feline nasal lymphomas. It is suggested that combined proliferation and apoptosis indices can provide a more accurate prognostic assessment, as reported in another study on humans [35]. However, another study used Ki-67 as the sole predictor for treatment outcomes [18]. While a relationship between cell proliferation and prognosis has been proposed, further research with a larger sample size of feline nasal adenocarcinoma cases is needed to clarify the underlying mechanisms.

In terms of treatment protocols, the results suggest that hypofractionated RT may still be beneficial for high Ki-67 tumors, even without full acceleration. Previous studies have highlighted the advantages of accelerated protocols (e.g., multiple fractions per day), which effectively reduce the interval between treatments and mitigate repopulation in rapidly proliferating tumors [18]. In contrast, the current study used a protocol with larger fractions over a less condensed timeframe. While this approach may not fully counteract tumor proliferation, the high dose per fraction likely contributed significantly to tumor control, partially compensating for the slower treatment pace [36]. Hypofractionation can be effective under specific conditions, particularly for tumors with high proliferative activity, as indicated by elevated Ki-67 levels. However, these findings do not undermine the potential advantages of accelerated protocols in such scenarios. Comparing hypofractionation with acceleration in the context of Ki-67 levels provides a valuable direction for future research to optimize strategies for managing rapidly proliferating tumors.

Based on the current results, 10 cats had initial leukocytosis before treatment. The causes of leukocytosis could include inflammatory conditions, glucocorticoid-associated factors, catecholamine-associated factors, or neoplasia [37]. Among these, five cats exhibited extremely high leukocytosis (range: 43.36–117.73 × 10^3^/μL), coupled with neutrophilia. In human studies, initial leukocytosis is common in patients with solid tumors, with reported incidence rates of 4–25.6%, often accompanied by neutrophilia [38]. Frequently, leukocytosis or neutrophilia is considered a poor prognostic factor in various cancers, including nasopharyngeal carcinoma [39], resected oral squamous cell carcinoma [40], metastatic colorectal cancer [41], lung cancer [42,43], gastrointestinal stromal tumors [44], and renal cell carcinoma [45]. Notably, we found that WBC decreased significantly (*p* < 0.001) after treatment. This reduction may have been due to the effects of anti-inflammatory drugs, antibiotics, or RT. In humans, several mechanisms have been proposed to explain the anti-inflammatory effects of treatment, including improved blood perfusion, the release of various cytokines and enzymes, modulation of molecule expression, effects on the local autonomic nervous system, innate immune system regulation, and changes in pH values [46,47]. These factors may contribute to reducing inflammation and, subsequently, WBC levels in the treated cats. Further research with larger sample sizes is needed to explore hematological parameters as potential prognostic indicators in feline nasal tumors. Additionally, further studies should investigate the underlying causes of WBC changes during treatment to better understand their clinical significance.

The current study had limitations due to its retrospective nature, as complete follow-up data were not available for all cats—such as clinical signs, imaging, full staging work-up, and necropsy findings. In addition, the relatively small sample size limited the statistical analysis. Furthermore, deaths of cats with undetermined or unspecified causes were attributed to nasal adenocarcinoma. Additionally, small biopsy specimens taken from large tumors may not fully represent the entire tumor, as noted elsewhere [48]. Moreover, factors such as histological differentiation, local tumor spread, and the presence of regional or distant metastases also play critical roles in determining overall prognosis, although no cat in our study had metastasis. A larger cohort study on feline nasal adenocarcinoma may be necessary to address these limitations. Future studies could benefit from a multi-faceted analysis that includes these additional prognostic indicators to provide a more comprehensive evaluation of treatment outcomes.

## 5. Conclusions

This study demonstrated that cats with a favorable response (CR or PR) had significantly longer survival times (1055 days) than those with a poor response (SD or PD) (369 days). Cats with high Ki-67 levels had a significantly longer MST than those with lower levels (1055 days vs. 256 days, respectively). Additionally, we observed that WBC was higher before treatment compared to after; however, there was no correlation between WBC and survival time. Our results suggested that Ki-67 may be a potential prognostic factor for feline nasal adenocarcinoma. Further studies with larger sample sizes are needed to confirm these findings and to enhance understanding of treatment benefits.

## Figures and Tables

**Table 1:** Patient characteristics of 19 cats with nasal adenocarcinoma.

Variable	Category	Number	%
Gender	Male	8	42.11
	Female	11	57.89
Reproductive status	Neutered	13	68.42
	Intact	3	15.79
	Unknown	3	15.79
Age (years)	≤10	12	63.16
	>10	7	36.84
Body weight (kg)	≤4.27	10	52.63
	>4.27	9	47.37
Breed	DSH	16	84.21
	Scottish Fold	2	10.53
	Persian	1	5.26
Retroviral status	FeLV antigen-positive	2	10.53
	FeLV antigen and FIV antibody-negative	13	68.42
	NA	4	21.05
Clinical signs	Sneezing	12	63.16
	Epistaxis	13	68.42
	Facial deformity	13	68.42
	Nasal discharge	7	36.84
	Dyspnea	5	26.32
	Ocular discharge	3	15.79
	Upper respiratory noise	3	15.79
	Submandibular lymph node enlargement	3	15.79
Tumor stage	T2	1	5.26
	T3	8	42.11
	T4	10	52.63

FeLV, feline leukemia virus; FIV, feline immunodeficiency virus; DSH, domestic shorthair; NA, not available.

**Table 2:** Comparison of hematological and biochemical results before and after treatment.

Variable	Before	After	*p*-Value	Reference Range
HCT (%)	32.6 (27.30–46.30)	35 (25.30–38.30)	0.605	30–45
WBC (×10^3^/μL)	19.36 (8.83–117.73)	14.39 (5.8–37.92)	<0.001	5.5–19
sCr (mg%)	1.61 (1.21–2.58)	1.67 (1.16–2.89)	0.865	1.0–2.2
ALT (IU/L)	41 (25–163)	46 (22–83)	0.518	28–76

Results are expressed as median (range). HCT, hematocrit; WBC, white blood cell count; sCr, serum creatinine; ALT, alanine aminotransferase.

## Data Availability

The data presented in this study are included within the article. The raw data supporting the findings of this study are available from the corresponding author upon reasonable request.
